# A Novel Resource Management Method of Providing Operating System as a Service for Mobile Transparent Computing

**DOI:** 10.1155/2014/153847

**Published:** 2014-04-23

**Authors:** Yonghua Xiong, Suzhen Huang, Min Wu, Yaoxue Zhang, Jinhua She

**Affiliations:** ^1^School of Information Science and Engineering, Central South University, Changsha 410083, China; ^2^School of Computer Science, Tokyo University of Technology, Hachioji, Tokyo 192-0982, Japan

## Abstract

This paper presents a framework for mobile transparent computing. It extends the PC transparent computing to mobile terminals. Since resources contain different kinds of operating systems and user data that are stored in a remote server, how to manage the network resources is essential. In this paper, we apply the technologies of quick emulator (QEMU) virtualization and mobile agent for mobile transparent computing (MTC) to devise a method of managing shared resources and services management (SRSM). It has three layers: a user layer, a manage layer, and a resource layer. A mobile virtual terminal in the user layer and virtual resource management in the manage layer cooperate to maintain the SRSM function accurately according to the user's requirements. An example of SRSM is used to validate this method. Experiment results show that the strategy is effective and stable.

## 1. Introduction

Transparent computing was proposed in [1] as a novel pervasive computing mode that provides centralized services including operating systems with the following several critical characteristics: that is, lightweight terminal without installing any operating system in advance, computing in local terminal and storing in remote servers, and dynamic loading and running operating system and program from the network on demand [2, 3]. In addition, the mode has been partly implemented in PCs with x86 architecture in [4] providing users the abilities to obtain desired services.

As a combination of transparent computing and mobile computing, also benefitting from the ubiquitous mobile networks and advanced transparent computing technology, mobile transparent computing enables the users to use a variety of operating systems on their own devices and experience massive cross-operating system applications while keeping all these data in the server so that the user can get a lot storage and ensure the safety of their data at the same time.

In such a mode that provides centralized storage and management of operating systems, user data, applications, and other resources and offers the users with unified, transparent, and ubiquitous service through network, all resources, including operating systems, are saved on the server of the transparent network permanently [5]. Obviously, the management and scheduling operations on the server for these networked resources will directly affect the performance of the whole system.

However, mobile devices are of several conspicuous characteristics like heterogeneous hardware architecture, more diversified OSs, more closely coupling between hardware and software, more unreliable network, and so on when compared to the PCs [6, 7]. Indeed, it brings many new challenges to improve and implement the methodology of transparent computing into mobile devices which have been increasingly popular throughout the world. The unreliable communication connection over wireless network makes it more difficult for real-time communication. Furthermore, the transparent network environment prone to lots problems: for example, a large number of users request the same resource simultaneously, network outages during the execution process of applications, resource conflict, and so on.

In order to provide trusted and efficient services to the end users, an effective resource management method that provides not only on-demand resource in a frequently changed mobile network but also seamlessly combination between heterogeneous hardware and software programs (including operating system) is vital.

For the purpose of sharing resources and running multiple OSs on different devices, the use of virtualization technology is suitable for mobile terminals where different OSes are hardly supported due to the closely coupling between hardware and software. QEMU (quick emulator) is a hosted hypervisor that performs hardware virtualization, which can be used to provide virtual hosting of several virtual computers on a single computer. The decoupling between physical mobile hardware and mobile OS together with other services provided by the QEMU hypervisor enables resources and services sharing among multiplatform even with different instruction sets, including x86, MIPS, ARM, PowerPC, SPARC, ETRAX CRIS, and MicroBlaze. This decoupling also yields affluent experience of various OSs, which enables a variety of unmodified guest operating systems to be run on the RAM by emulating it through dynamic binary translation.

Furthermore, for the purpose of discovering, organizing, allocating, and managing those shared resources and services for the MTC environment where the network connection is not that reliable and the location of mobile terminals are frequently changed, the mobile agent [8] is introduced. The use of mobile agent benefits the on-demand resource and the streaming execution in MTC, since the mobile agents are able to carry the network resources and store the state while automatically migrating from a network node to another. Besides, the agent decides itself where and when to migrate while performing the management functions, which means mobile agent can continue working even the device is not connected, thus to reduce the network usage.

In this paper, we introduce a mobile device suitable mode namely the mobile transparent computing (MTC) by extending the methodology of transparent computing in desktop terminals, and then analyze the requirements for resource management in MTC. Specially, we present a kind of shared resources and services management (SRSM) strategy for MTC based on mobile agents and virtualization technology. The proposed approach is designed in a three-layered architecture: that is, the user layer that communicates with the network and submits the requirements on behalf of a user; the manage layer that acts as the core manager that discovers and allocates resources in SRSM; the resource layer that is responsible for shared resource and service storage in the transparent server. By applying the QEMU virtualization technology on the user layer, it works well under multiplatform and multidevice environment in MTC. Furthermore, the use of mobile agent in the manager layer deals with the problem of mobility and unreliability of wireless network connection. Through interactions of mobile agents in the manging layer, mobile users are able to get their desired resources and services from anywhere and possibly via heterogeneous mobile devices.

The remainder of this paper is organized as follows.  Section 2 describes the related works on resource management method. In Section 3, the novel concept of MTC is given and the requirements for managing service in MTC environment are listed.  Section 4 presents the SRSM method that we have developed using QEMU and mobile agent in MTC environment. Implementation and results are discussed in Section 5. Finally, Section 6 includes a discussion of our conclusions and projected future work.

## 2. Related Works

In MTC, Network resources are distributed, heterogeneous, autonomous, and dynamic, which make resource management, task response, and performance monitoring the core issue of MTC. Users simply submit resource requests, while resource searching, allocation, monitoring, and other operations all will be completed by the server. Existing related works on resource management could be categorised into two aspects: one is considered from the server terminal and the other is from the mobile device.

On resource management of server terminal, cloud based service management has been an important hot research topic in recent years. A survey [9] conducted by Rasmi and Vivek summaries some resource management techniques in cloud environment. The virtualization is a key technology in enabling managing the resources in cloud-based solutions [10]. In practice, the cloud computing is done with virtualization of some sort such as Amazon EC2. The virtualization provides a security mechanism in clouds and may make it possible to capture valuable information in ways that are implausible without VMs [11]. A recent study proposed a framework that allows virtualization-supported cloud protection across physical hosts over the Internet. It is based on the security extensions of the Linux Kernel Virtual Machine (KVM Qumranet), thus to increase the security of cloud resources [12]. Moreover, within the virtualization concept, customers are able to get their desired resources among heterogeneous devices [13]. Existing approach [14] monitors guest domain and analyzes runtime information for automating resource allocation based on XEN virtual machine.

Most methods of managing resources in cloud cannot be directly applied to mobile computing because mobile computing contains wide heterogeneity of mobile devices, strong coupling between hardware and software, unstable wireless link, etc. Considering the conspicuous characteristics of mobile devices, existing methods [15, 16] also address the issues of resource management for embedded systems, which preserves system characteristics despite the presence of dynamic resource allocation. Mobile agent is used to address the problem of unreliable wireless network connection when dynamically allocating resources to wide heterogeneity of portable access devices [17, 18], for it assures the resource discovery and performance management in the context of service-oriented networks. In the previous study [19], mobile agent is designed into a middleware for it does not need continuous network connectivity while performing tasks. And in [20], the mobile agent paradigm was integrated with genetic algorithm to provide efficient resource allocation in a multioperator networking environment. Besides, the mobile agent is often used in solving the problems of network resource scheduling in clouds [21, 22] for their ability of cooperation with each other and adaptively migrate among the network nodes. The agent based approach can effectively perform basic management of network resources. That is why the mobile agent is suitable for resource management in mobile network.

However, the abovementioned solutions on server terminal and mobile device are not suitable and effective for resource management in MTC environment, for the special characters of resources in MTC are not taken into account, such as allocating the OS image, supporting multiplatform, and computing in the end-device. Therefore, this paper proposes the resource management method using virtualization technology and mobile agents for mobile transparent computing.

## 3. Framework of Mobile Transparent Computing

This section provides a basic idea of mobile transparent computing and presents some requirements of shared resources and services management across multiple mobile devices in MTC environment.

### 3.1. Mobile Transparent Computing

As previously noted, the MTC is defined as a mobile device suitable for computing mode consisting of mobile computing and transparent computing.

#### 3.1.1. Transparent Computing

In recent years, transparent computing [1] was proposed as a new pervasive computing with the goal of providing users with transparent services. Different from the remote computing and remote storage in cloud computing, the programs in transparent computing are executed locally while stored in the remote server. In addition, in transparent computing, the operating system is also regarded as a resource that can be loaded and used when needed, thus to form a new service model: operating as a service (OaaS). That is to say, users only concern whether they can get the service or not, but no need to know the underlying details of the required services.

The transparent computing paradigm consists of transparent clients, transparent network, and transparent server. In particular, the clients are not proposed to have any operating system or application software on it for having few or even no local storage. Numerous OSs and applications are stored in the remote server and can be streamed to the clients via transparent network. When a task processed on client is completed, all data and information will be submitted to the transparent server (TS).

As all resources are stored in the transparent server, whether they can be managed and scheduled in a proper way will directly affect the performance of the whole system. Ning and Sun have developed a remote resource management method based on XEN virtual machine [14] to collect the state of resources and applications on the device and offer resource management and storage for PC in transparent computing paradigm. Within the mechanism with a Xen hypervisor running on PC that not only conducts local monitoring, but also provides sufficient isolation between host OS and client OS that is loaded from a remote server, and the manager agent that works in each client is able to gather information and generate messages to the manager center, which runs in manager server and performs resource scheduling and service assurance.

However, the above method for resource management did not consider the situation of mobile devices, such as closely coupling between hardware and software, wide heterogeneity in instruction sets, and unreliable wireless access in portable mobile devices. In order to discover the on-demand resources and manage a consistence of shared resources for various mobile users, we develop a mobile transparent computing architecture by extending the transparent computing to mobile devices.

#### 3.1.2. Mobile Transparent Computing

**Figure 1:**
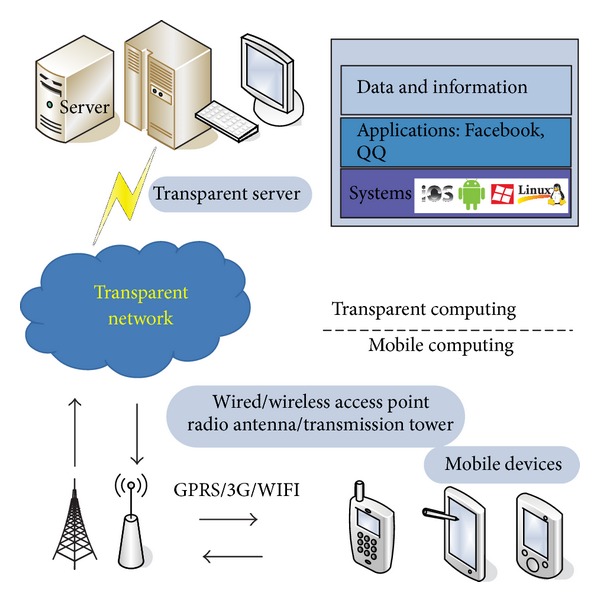
Framework of MTC.

As a combination of transparent computing and mobile computing, the framework of MTC is shown in Figure 1. Under the MTC circumstances, mobile OS (IOS, Android, Windows Phone, etc.), mobile applications (Facebook, mobile IMs, mobile game, etc.) and user data are all stored in the transparent server and are regarded as sequences of execution blocks rather than a whole, while only few communication protocols and management processes, which are used to interact with users and connect with a hot spot or a base station by 3G, WIFI, or GPRS, are left on the device.

When powered on, the mobile device will connect to the server through transparent network and request the least needed blocks to be executed from the server. Thus all execution blocks of any programs, namely, OSes, applications, and so on, can be streamed to mobile device. When execution is finished, the results will be saved to the TS, which can be accessed and continued by any mobile device the next time you start it.

Since resources are stored in remote server, mobile users can load and save as much applications and data as possible without worrying that the limited storage of his own device will be taken. Moreover, in this way, personal application programs and data are independent of the device, wherefore acquiring any requirement at any time and on any device anywhere is available in ideal conditions including sufficient network width and homogeneous hardware architecture. Yet, this calls for a remote resource management method that provides shared resources and services among heterogeneous devices in a mobile network.

### 3.2. Resources in Mobile Transparent Computing

In general, the resources we mean in grid computing or cloud computing including physical resources (CPU, memory, storage, network elements, actuators, and so on) and logical resources (including operating system, energy, network throughput/bandwidth, and so on) [23]. In the process of handling a cloud service, the manager should attribute the job (applications or other services) to a relevant server to execute and reply with the computing results. The problem of resource management mainly refers to how to manage these computing resources that can complete the computation tasks of cloud service users and, at the same time, meet the requirement of service provider, whether to minimise the total cost or maximize the CPU utilization or some other optimization goals.

While being in mobile transparent computing, the resources merely refer to application resources including OS, applications, user data, and customized services; thus the process of handling a transparent service can be simplified as the manager searching in the server of the requested resources and deliver it to the corresponding user through network. These applications and services are transported to and executed on the mobile device, rather than on the server as in cloud computing. Therefore, the management problem comes as how to manage these application resources in a safe and efficient way and, simultaneously, reply more mobile users as soon as possible.

The resources and services in MTC, which are managed in the transparent server and can be accessed by any mobile device through network at anytime and anywhere, are virtual disk files (VDF) to mobile users that can be divided into the following three types according to the service mode.

Public VDF is shared by all mobile users in the MTC environment, including mobile OS kernel images, root file systems, various applications, and all common services. These files can be accessed by any user.

Private VDF, owned by the particular user with credential, is users' personal resources, which include user data (photos, contacts, messages, etc.), customized applications, and services.

Mixed VDF is owned by user groups, such as a company and a school. All users belonging to this group are able to get the shared resources and services, which mainly include group shared files, news, and software.

In MTC environment, it is vital to gather the overall resource information in a wireless network and manage all these resources in the server.

### 3.3. Requirements for Resource Management in Mobile Transparent Computing

As a core function required for any manmade system, resource management affects the three basic criteria for system evaluation: performance, functionality, and cost. In the MTC environment, where changes are frequent and unpredictable, an efficient resource management strategy is needed to provide continuous service and performance guarantees. Indeed, the proposed SRSM aims to provide adequate solutions to the host of shared resource and service management policies you have to enforce.

**Figure 2:**
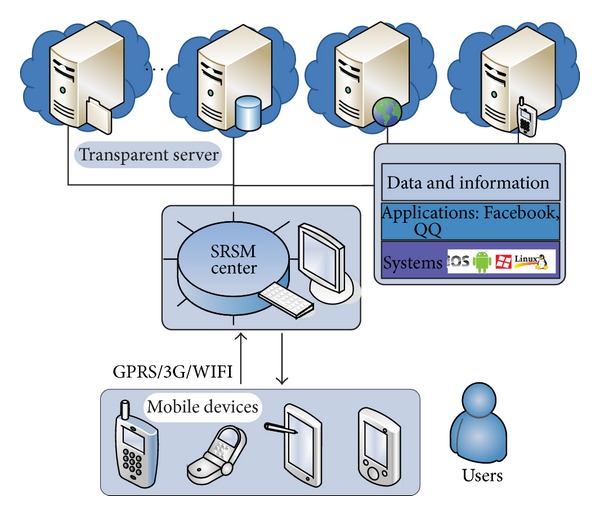
SRSM in mobile transparent computing.

The SRSM strategy is designed to manage and schedule these virtual disk files in a reliable, efficient, and proper way.  Figure 2 depicts the MTC environment with the SRSM center as the core manager. Therefore, the important characteristics and requirements for resource management in the MTC environment should be cleared, which are as follows.

#### 3.3.1. Making Resources and Services Available on Demand

MTC aims to provide on-demand resource and service, which means the SRSM has to monitor and listen all requests in the transparent network, then searches, organizes, and allocates the resources that are demanded.

#### 3.3.2. Sharing Resources and OSes among Multiplatform Devices

Mobile devices vary a lot in hardware architecture, and the coupling between physical hardware and software makes it difficult to run multi-OS and cross-platform applications on a particular mobile device. However, the SRSM we proposed is supposed to support resources (including OS and application) sharing among multi-platform devices. What's more, the principle that the client has no preinstalled operating system locally in MTC makes it more challenging.

#### 3.3.3. Dynamic Scheduling and Streaming Execution

In order to run huge applications even with limited CPU capability and storage, the MTC mechanism divides the resource into small streaming blocks and is steamed to mobile device when and only it is called. The SRSM center is responsible for dynamic scheduling of those blocks rather than the entire application or other resources, to complete the needed service.

#### 3.3.4. Flexible Authority Provisioning to Different Resources and Services

As mentioned before, different users and groups own different authorities to the shared VDF in the server; that is, all users share the same public VDF, while each user owns a private VDF. Some users may in the same group, thus all users in that group are able to use and share a mixed VDF. A flexible authority provisioning is needed to ensure the system performance in a desired pattern.

For the purpose of meeting these requirements and addressing these challenges, the SRSM is discussed and designed in the following section.

## 4. Resource Management Using SRSM

### 4.1. Shared Resources and Services Management (SRSM) Overview

**Figure 3:**
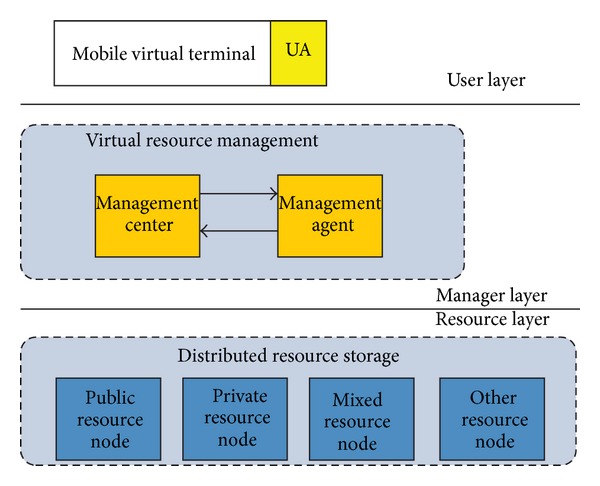
Architecture of SRSM.

Implementing the SRSM (shared resource and service management) to support MTC environment requires consideration of virtualization technology and extending of the mobile agent architecture. In this section, we show how, by using the technology of virtualization and mobile agents, the SRSM is designed with the goal of performing the basic management operations of network resources. We have developed an agent based resource management method, which is organized in a three-layered architecture as Figure 3 shows, namely, the user layer, manager layer, and resource layer.

At the highest level of the SRSM architecture is the user layer. With QEMU running on various mobile devices and performing device virtualization, the user agent (UA), which also runs in the user layer on behalf of the user, is capable of acquiring the resource information and requirement of various devices with different hardware architectures. The mobile agent is adopted in the manager layer that offers centrally management of all distributed resources and services which are called virtual resource here.

The manage layer lies in the middle acting as the core manager and is composed of a manager center and several manager agents, by cooperation of the manager center and manager agents, the management and allocation of all virtual resources and services on transparent server can be achieved. With the mobile agent technology, the manager layer is able to provide the interfaces to network service and interact with user agents.

At a lower level of the architecture, the resource layer centrally stores all resources such as public VDF and private VDF in MTC resource nodes. The resource layer, as a provider of all resources and services, should shield the details of all resources to the manager layer and provide appropriate services to the user layer.

In particular, the SRSM three-layered architecture consists of three parts, namely, mobile virtual terminal, distributed resource storage, and virtual resource management. Each of the three parts lies on one layer and will be discussed in the following sections.

### 4.2. Mobile Virtual Terminal

**Figure 4:**
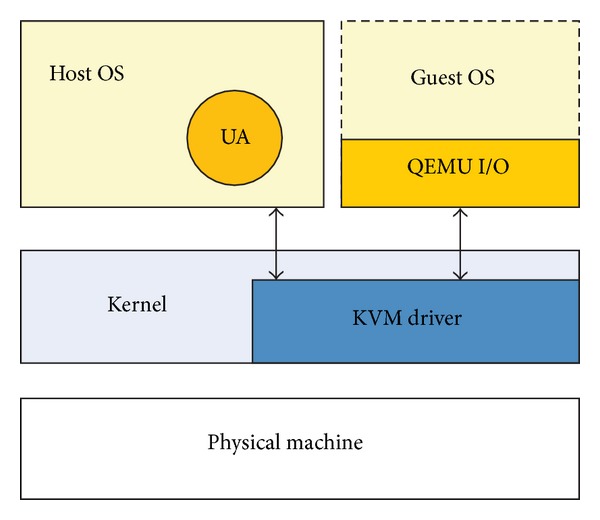
Mobile virtual terminal.

The mobile virtual terminal (MVT) is on the user layer, which is distributed on multiple mobile devices and supports the SRSM services at different locations, possibly via heterogeneous mobile devices by using the QEMU virtualization technology.  Figure 4 reveals the composition of mobile virtual terminal.

The decoupling between physical machine and OSs or software by using the QEMU hypervisor makes it possible to support more than a particular OS on multiple mobile devices, for the QEMU can boot several OSs, such as Linux, Windows, and Mac OS, on different instruction sets.

Within the QEMU, together with a KVM (kernel based virtual machine) driver, which can drive some physical device and present as a whole device to the guest OS, the mobile device is virtualized. We run a host OS on it, and then any type of OS can be streamed from the SRSM server and run as a guest OS on this mobile virtual terminal almost. The interactions with guest OS and host OS are done through the QEMU, thus to enable normal use of guest OS. We run a user agent (UA) on the host OS to communicate with the SRSM center and redirect the I/O requirement from MVT to a remote server. MVT benefits greatly from a UA-based implementation because of user agents receiving, analyzing, and processing the requests of resource and services transmitted from the user layer and redirecting those requests to the SRSM center on behalf of the user without showing the details of processing those requests in manager layer. In addition, the UA is responsible for consulting with the manager layer thus to offer the MVT with most satisfying resources and services.

The resources and services are streamed from the manager layer and executed on the MVT. During the whole execution, the UA provides monitor services that gather the state information of the ongoing services. The UA in MVT can save the state of the application process which will be synchronized to the SRSM server once the connection between UA and manager layer is alive. As a result, any MVT with the completely same authority should continue to access the needed network resources and services independently of its location. This kind of service migration from one mobile device to another embraces a new era of multidevice support in MTC environment.

### 4.3. Virtual Resource Management

**Figure 5:**
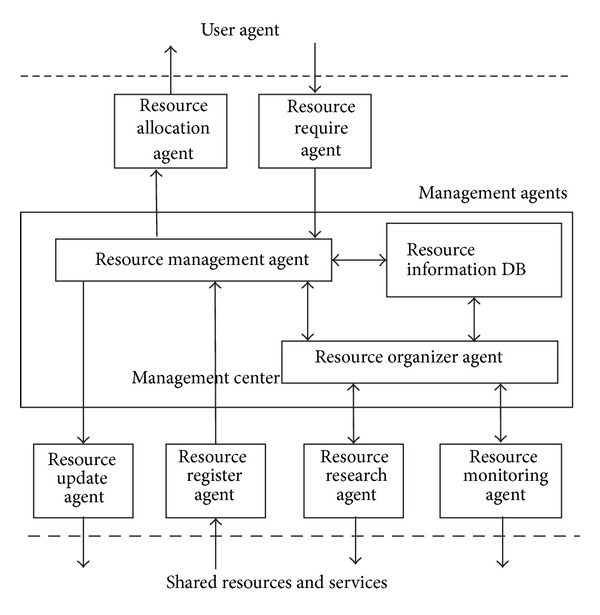
Virtual resource management.

The virtual resource management (VRM) is designed based on the mobile agent technology. As Figure 5 shows, the manager center (MC) and several manager agents make up the VRM in the manager layer, in SRSM. The VRM is in charge of managing and allocating the available virtual resources and services. For the MVT, VRM acts as a server to acquire the request from the user and schedule the on-demand resources and services dynamically. At the same time, VRM provides centrally maintenance of the properties, location, tags, and other information of all available resources and services for the resource layer. The cooperation with the MC and MA allows the SRSM to perform even complex management in MTC.

The mobile agent based VRM can be used to collect and manage all the shared resources and services required. The cooperation of MC and MA facilitates the implementation of SRSM in an efficient way and helps overcome some of the challenges we described previously, such challenges may involve in dynamic scheduling and streaming execution, resources available on distributed mobile devices in MTC environment under consideration. In the following, we focus on the functions of the MC and MA which are all based on the mobile agent technology.

#### 4.3.1. Manage Center (MC)

As the core of the VRM, the MC is in charge of controlling and coordinating the manager agents in the entire system to accomplish resource management in MTC. The MC is composed of three functional modules: resource management agent, resource organizer agent, and resource information database. Each of the three modules has different functions in resource manage layer.

The management agent is responsible for the core manager issues such as MA coordination, resource aggregation, and configuration and, most importantly, the centralized control of SRSM. The organizer agent is designed to cooperate with the management agent. It can help dealing with the issues like resource information collection, resource integration, and organization. The resource information database is a set of resource information that records the properties, locations, tags, and other information of the corresponding resources and services.

For the purpose of matching the correct resource efficiently, a unique name and the corresponding map of its physical location are also given in the database. All these information are collected by different manger agents from the distributed resource layer and are organized by the organizer agent and then sent and saved to the information database.

The information database is shared and accessible by both of the management agent and organizer agent, for they need to know the tags, contents, locations, and concrete information of all the resources that are managed and stored in the server.

When a resource request comes from the user layer, is delivered by the user agent, and is received, the management agent will first analyze the request by resolving the properties, tags, and other concrete information of the resource or service that is requested. Thereafter, the management agent will search for the corresponding information on the resource information database and tell the organizer agent to search for the requested resources in physical resource layer according to the location and other information that are matched and approved by the management agent. By arranging some manager agents to search for the needed resource and service, the organizer agent is able to gather and organize those resources and services, which will be transferred to the resource management agent and finally allocated to the mobile device through the MTC network.

#### 4.3.2. Manager Agent (MA)

In order to perform the management of virtual resources and services under the MTC environment, we develop the following basic types of mobile agents to work together under the control of the manager center and accomplish about the resource register, research, monitoring, allocation, and other actions in SRSM.

*Resource Register Agent.* When a new resource or service is added to the resource layer, the register agent (RGA) will register the resource to the manager center. In addition, the RGA should be able to establish an available list that described the information to locate massive resources. The information will be saved to the resource information database, which provides a reasonable basis to the subsequent resource research and scheduling.

*Resource Require Agent.* The required agent (RQA) receives the resource and service requirements from various remote UAs. The resource allocation plan, which will soon be sent to the MC to be processed, is specified by the RQA according to user authentication, user requirements, and allocation rules. The RA, during its working process, interacts with the distributed user agents for collecting resource request submitted by them.

*Resource Research Agent.* The research agent (RSA) realizes the functionalities of providing an efficient and rational resource research approach. In general, the RSA searches the resource layer for the required resources and services according to the tags provided by the manager center and then passes the research results to the organizer agent, to accomplish a research process. If the needed resource is not found, a report with the information of resources not found will be produced by the RSA and be passed to the database through the organized agent. Thus to prevent researching this unfound resource repeatedly when another request comes and asks for the same resource, until this exact resource is registered with its information saved to the resource database.

*Resource Monitoring Agent.* The resource monitoring agent acts as a monitor that announces the status of resources and other mobile agents and then produces a corresponding report that contributes to the control and regulation actions of the management agent. Moreover, it also estimates the delay time of a resource request, which might be useful and humane for sharing resources and services among remote mobile devices.

*Resource Allocation Agent.* The allocation agent (ALA) is responsible for resource and service allocation under control of the management agent in MC. The management agent will produce a resource allocation strategy, according to which, the ALA assigns the needed resource to the corresponding user. Through interactions with UA, the ALA is able to transfer the resource, OS, and other services to the user or, more exactly, the MVT.

*Resource Update Agent.* As noted previously that all the updates, modifications, and changes will be saved to the remote transparent server, it thus highlights the necessity of updating the resource information in SRSM. The update agent (UDA) can update the changes to the resource layer under the guidance of manager center. In fact, when the user asks for some service, the contacts, for example, from network, they can get the contacts saved before through cooperation of MC and other mobile agents. And if the user adds a new contact record to his contacts, this new record will be checked by the UDA and be written to the where the original contacts are. With the UDA, all the increased record of VDF will be updated to the resource layer. At the same time, the information of its change will be updated to the MC.

### 4.4. Distributed Resource Storage

**Figure 6:**
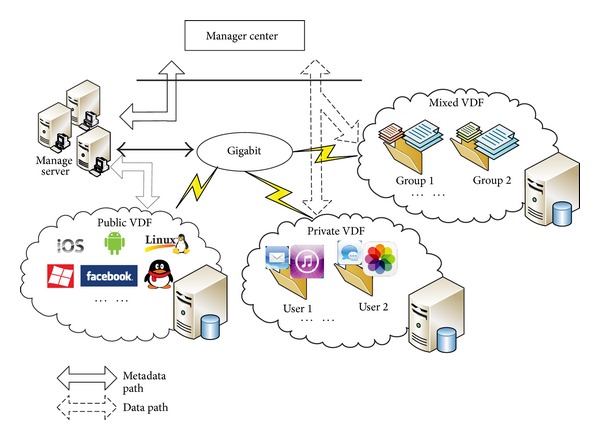
Distributed storage of resources and services.

In the three-layered architecture of SRSM, the distributed resource storage works on the resource layer, with the task of storing all shared resources and services. As Figure 6 shows, the resources and services are divided into public VDF, private VDF, and mixed VDF according to the service mode. Respectively, different VDFs are distributed and stored in a different server, that is, public resource node, private resource node, mixed resource node, and other resource nodes, which facilitates the management a lot.

The distributed VDF is entity that can be accessed and updated by the owner during a period. Any user in the MTC transparent network can access the public VDF, while only the SRSM manager center is allowed to change it. For the private VDF, which is stored in private resource node, it is accessible and changeable by the user with the corresponding authentication. In the same way, the mixed VDF is stored in mixed resource node and are accessible by the relevant group members. All distributed resource nodes in SRSM are connected through Gigabit ethernet connection.

For the purpose of providing fast and efficient service to users in MTC, the beyond band data transfer mode is taken. As the visit frequency and times of public VDF are far more than that of other files, we name the public VDF as metadata and separate the metadata from file data.

There are dedicated servers that specially manage public VDF and provide a straight access for metadata. In addition, a scheduler will be used to balance the load of these management servers, which is also in charge of deciding the distribution of activated metadata between various servers and coordinating their operations. While the less visited file data (private VDF and mixed VDF) such as messages and photo are managed globally by SRSM.

The distributed resource storage is the provider of shared resource and services, all the transparent services that are provided to the mobile users will finally be saved to this resource layer which seems like a data center that offers various resources and services to the MTC users.

## 5. Implementation and Discussion

**Figure 7:**
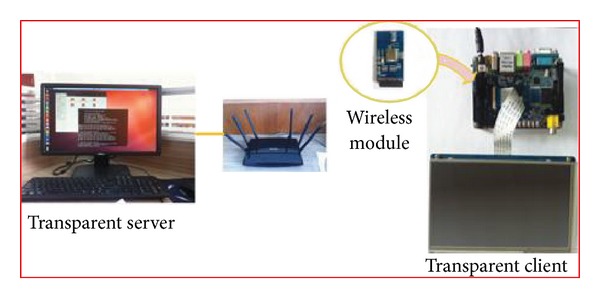
Experimental environment.

In this section, we implement the proposed SRSM on some real mobile devices and demonstrate the field experiments.  Figure 7 shows the real experimental environment. We use a demo tablet board with the MVT technology as the SRSM mobile device and an ordinary PC equipped with Ubutu12.04, together with SRSM center and shared resources and services on it as the SRSM server. The sever and client are connected through a wireless module (marvell 8686).

Users can create user agent in the mobile device and deliver the resource requirement to the network, while other mobile agents dispatch it to relevant node to get the desired resources, whether mobile OSes or applications. Based on the SRSM strategies we described in the previous sections, we design three situations that our SRSM may be most helpful in the MTC environment.

### 5.1. Operating System as a Service

As we know, the OS in MTC is regarded as a service and stored in the SRSM management server rather than directly installed in the mobile device, which left the device almost a bare hardware without any mobile OS or data on it.

First, we confirmed that the OSs are stored in the management server rather than the mobile device and the service entries are disseminated in the transparent network by manager agents. The tablet is equipped with our user agent, through which the desired OS can be loaded as a service from the management server.

**Figure 8:**
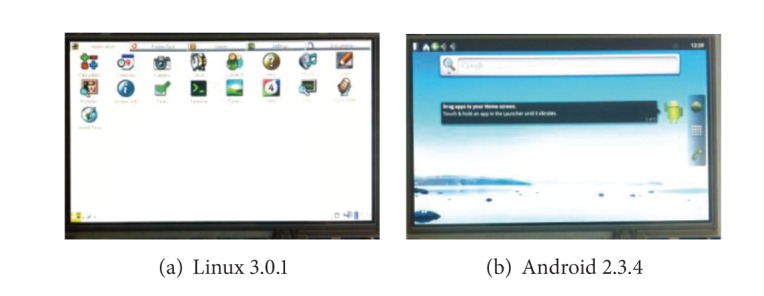
Multi-OS on-demand with SRSM.

In order to verify the efficiency of the SRSM strategy, we try to load the Linux 3.0.1 and Android 2.3.4 in succession to the tablet through network.  Figure 8 shows that we are able to load and boot Linux (Figure 8(a)) and Android (Figure 8(b)) through the transparent network thanks to the SRSM strategy.

**Figure 9:**
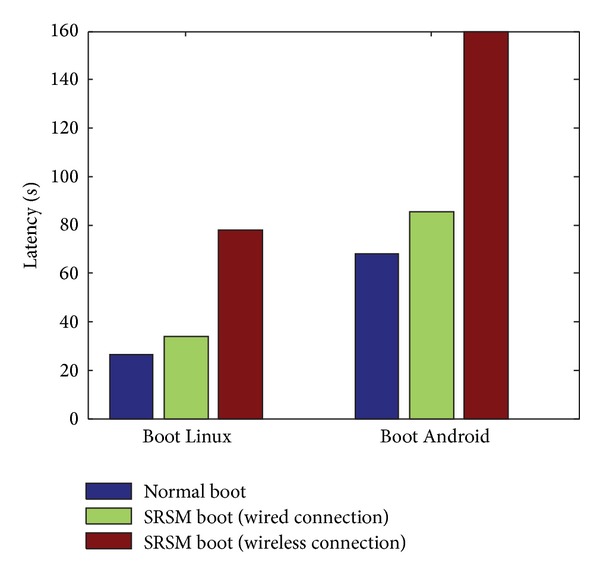
Multi-OS on-demand with SRSM.

Furthermore, we compared the OS booting time of normal boot and net boot within SRSM strategy. The local boot time of Linux is 26.5 seconds, and 68 seconds for Android. The latency (as shown in Figure 9) of net boot under wired connection is close to that of normal boot with the time of 33.9 s (Linux) and 85.5 s (Android). While being under wireless condition, the latency is almost two times of wired connection with the time of 78 s (Linux) and 160 s (Android), yet still acceptable.

### 5.2. Centric Resource Management for Local Execution

**Figure 10:**
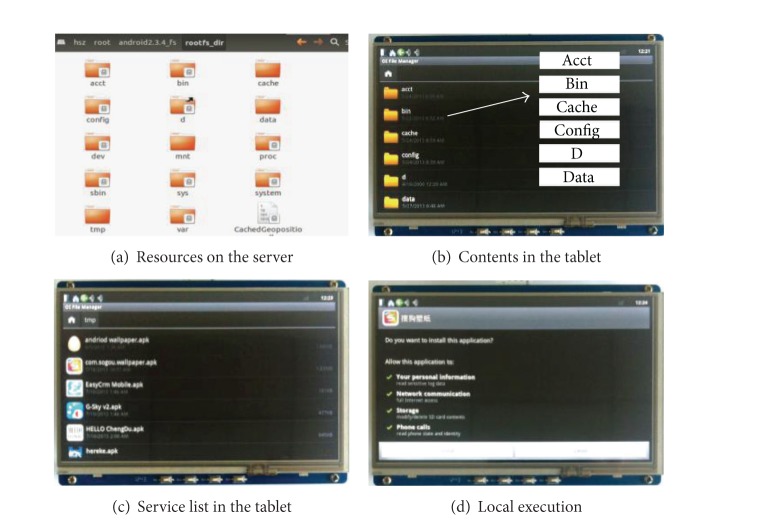
Centric management.

Besides the mobile OS, all the public resources, private resources, and mixed resources are centrally managed and stored in the SRSM manager center. All these resources are presented to mobile users as VDF and can be accessed as normal disk file.  Figure 10(a) demonstrates that all resources that are stored in the resource layer, which is located in the SRSM server as we stated before, can be accessed by the mobile agent via transparent network. When we powered on the mobile device and the contents we got in the tablet are exactly the same with that on the SRSM server as Figure 10(b) shows.

With the centric management of the manager layer in SRSM, these centrally stored resources are available on demand. In the field experiments, after all network resources were updated and prepared, the user agent in the demo tablet board requested the service based on the service list (as shown in Figure 10(c)) that was shared by all mobile users and was disseminated by manager agents. Through the interactions of user agents and manager agents, any service provided by the SRSM server can be streamed to the tablet and executed on it. In Figure 10(d) we installed an application from the transparent network. This application runs smoothly and stably in our mobile device.

Tables 1 and 2 illustrate the performance of streaming execution of some applications. We compared the latency of normal device and mobile virtual terminal within the proposed method under transparent environment in the following three aspects: file coping, file downloading, and application launching. The results show that all these operations can be done as normal both in Linux and Android. Though the response time is not that ideal, it is yet still acceptable. As all these applications are streamed from the network when called, the response time mainly depends on the network condition that is why mobile virtual terminal spends more time when executing a program. The same conclusion can be drawn from file coping and file downloading.

However, it is worth mentioning that, with SRSM in MTC, all the data, applications, and customized services that the user kept in the tablet will be saved to the server as private VDF, rather than to the mobile device, thus to enable any device with the same authentication accessing the same customized services and provide a limitless storage for users. In addition, the security of the customized services and data will be enhanced for the separation of mobile device and user data ensured the safety and entirety of all resources without being lost or destroyed in the consequence of unexpected accidents on the mobile device, such as hacking, damaging, loss, and so on.

### 5.3. Sharing Resources among Different Devices

Under the structure of distributed resource layer in SRSM, the public VDF is unified managed by the SRSM center. Thus, the users do not have to worry about the boring issues like firmware updating, OS changing, software updating, and managing. When a mobile device is connected to the transparent network, all these public VDFs will be able to be loaded totally free. Once a user account is created, all the resources that we saved from any device will be stored to the SRSM server as private VDF and can be synchronized to any other device with the same authentication.

For example, it is possible for one to take photos with his mobile phone when he is outside while sharing these beautiful photos with his family from a tablet or another device whatever is convenient, when he is back home. It is the same with the applications or other services, for if one installs an application to his private VDF from one device, he will be able to use this application from any mobile device whenever he is in connection with the transparent network.

With the SRSM strategy, we are able to share resources and services among different devices even with heterogeneous hardware.

**Figure 11:**
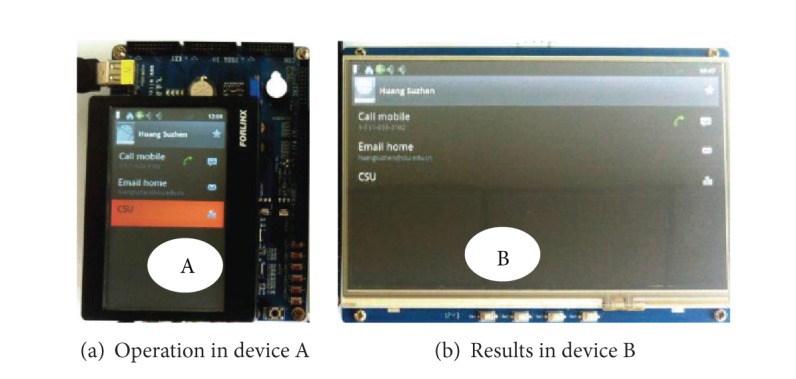
Resources in different devices.

In the example in Figure 11, we add a new member in the contact in device A, and then the newly added member can be stored to the private VDF in the server that can be accessed by another device; in this situation we name it device B. When device B is powered on, it is regarded as a MVT to the SRSM management server. If the contact is requested, the UA in device B will ask for the latest saved contact, which includes the newly added member in device A. The result is demonstrated in Figure 11(b), for the newly added member is accessible and editable by another device, which means we can share a same resource even with different devices no matter where we are or what architectures the devices are.

In short, the above experimental results show the effectiveness and feasibility of the proposed SRSM method. By combination of QEMU virtualization and mobile agents, the SRSM strategy succeeded in meeting the requirements of resource management for mobile transparent computing. It provides multi-OS support for the same hardware, centric resource management of all users, local execution of programs, and sharing resources among multiplatforms and multidevices. Though the performance of SRSM in our experiment is not that satisfactory, it indeed offers a new perspective of resource management under mobile transparent environment.

## 6. Conclusion

The transparent computing mode offers new challenges and opportunities for researchers interested in service providing and resource management in the remote server. In this paper, we introduced a mobile device suitable for transparent computing architecture in which mobile users are able to access the desired resources even including operating systems anytime and anywhere by extending the transparent computing mode to mobile devices. The typical features of mobile devices that set it apart from previous work include diversified hardware and OS, close coupling of hardware and software, and unreliable wireless network connection. Therefore, we proposed a novel resource management method that provides operating system as a service for the introduced mobile transparent computing based on the integration of QEMU virtualization and mobile agents technology.

With the QEMU running in the end device, all mobile devices, no matter what architectures they are, are regarded as MVT to the SRSM center. Thus to realize operating system as a service from two aspects: supporting different OSs and applications in the same hardware, sharing the same resources and services among different devices.

The use of implemented mobile agents is to prove the effectiveness of SRSM for solving some problems existed in mobile network. The mobile agent based on SRSM makes it easier to collect the information of the network resources and allocate the needed resources to the corresponding device within an unreliable wireless network connection.

As part of the research, we implement the SRSM in practical mobile transparent computing environment, and the results of a comprehensive performance analysis show that our proposed method is capable of providing operating system as a service, managing resources on the remote server, executing programs on the end device and sharing resources among different devices.

To further improve the performance of our method, the scheduling algorithm and authentication control are quite considerable. For a proper algorithm that decides resource dynamic scheduling will considerably reduce the responding time and operation latency in our work. So far, the presented work has not taken the situation that multiusers exist at exactly the same time into account which often occurs in facet. Therefor the authentication control in MTC is needed. Besides, we are planning to develop an algorithm to travel the mobile agents under the situation of overloaded network traffic in mobile networks.

## Figures and Tables

**Table 1:** Operation latency in Linux (seconds).

Operation	Normal device	Mobile virtual terminal
Copy a file		
5 M	2.18	3.43
10 M	3.98	6.99
50 M	18.16	28.26
Download a file		
5 M	7.27	9.90
10 M	14.19	19.13
50 M	71.69	101.01
Launch an App		
WEB browser (startup)	3.65	2.25
Snake (start game)	2.28	1.89
Video player	3.75	4.07

**Table 2:** Operation latency in Android (seconds).

Operation	Normal device	Mobile virtual terminal
Copy a file		
5 M	3.56	4.59
10 M	7.15	9.65
50 M	28.41	31.29
Download a file		
5 M	9.02	7.90
10 M	17.95	18.70
50 M	88.56	100.46
Launch an App		
WEB browser (startup)	4.94	6.91
Snake (start game)	1.91	2.50
WPS office (startup)	6.76	14.18
WPS office (open 1 M file)	9.31	18.43
